# Low Cost Sensors Based on SPR in a Plastic Optical Fiber for Biosensor Implementation

**DOI:** 10.3390/s111211752

**Published:** 2011-12-16

**Authors:** Nunzio Cennamo, Davide Massarotti, Laura Conte, Luigi Zeni

**Affiliations:** 1 Department of Information Engineering, Second University of Naples, Via Roma 29, 81031 Aversa, Italy; E-Mails: laura.conte@unina2.it (L.C.); luigi.zeni@unina2.it (L.Z.); 2 Department of Physical Sciences, University of Naples “Federico II”, Via Cinthia, 80126 Naples, Italy; E-Mail: dmassarotti@na.infn.it; 3 CNR-IREA, Via Diocleziano 328, 80124 Naples, Italy

**Keywords:** Surface Plasmon Resonance (SPR), Plastic Optical Fibers (POF), biosensors

## Abstract

This paper reports the fabrication and testing of two configurations of optical sensor systems based on Surface Plasmon Resonance (SPR) at the interface of a liquid sample and sandwiched structures realized starting from the exposed core of a Plastic Optical Fiber (POF). The proposed geometries have proven to be suitable for measuring the refractive indexes of liquids whose refractive index falls around 1.35. Furthermore, the proposed sensing head, being low cost and relatively easy to realize, may be very attractive for biosensor implementation.

## 1. Introduction

Surface Plasmon Resonance is known to be a very sensitive technique for determining refractive index variations at the interface between a metallic layer and a dielectric medium (analyte). SPR is widely used as a detection principle for many sensors operating in different application fields, such as bio and chemical sensing. In practical implementations, the biological targets are usually transported through a microfluidic system by means of a buffer fluid or a carrier fluid. In SPR sensors, the transducing media (ligands) are usually bonded on the metallic layer surface so that, when they react with the target molecules present in the analyte, the refractive index at the outer interface changes, and this change is detected by suitable optical interrogation. In the scientific literature, many different configurations based on SPR in silica optical fibers, are usually found [1]. In general, the optical fiber employed is either a glass one or a plastic one (POF). For low-cost sensing systems, POFs are especially advantageous due to their excellent flexibility, easy manipulation, great numerical aperture, large diameter, and the fact that plastic is able to withstand smaller bend radii than glass. The advantage of using POFs is that the properties of POFs, that have increased their popularity and competitiveness for telecommunications, are exactly those that are important for optical sensors based on optical fibers [2]. The advantages of POF sensors is that they are simpler to manufacture than those using silica optical fibers [1,3]. In the scientific literature there are only simple POF sensors based on laterally polished bent sections prepared along a plastic optical fiber [4,5]. In this paper two different POF sensor configurations are presented and experimentally tested. The classic geometry of sensors based on SPR in silica optical fiber is adapted and borrowed for Plastic Optical Fibers, so representing a simple approach to low cost plasmonic sensing. The planar gold layer and refractive index ranging from 1.332 to 1.418 are two good factors for forthcoming biosensors implementation.

## 2. Experimental Section

### 2.1. Background

In the optical phenomenon of Surface Plasmon Resonance, a metal-dielectric interface supports a p-polarized electromagnetic wave, namely Surface Plasmon Wave (SPW), which propagates along the interface. When the p-polarized light is incident on this metal-dielectric interface in such a way that the propagation constant (and energy) of resultant evanescent wave is equal to that of the SPW, a strong absorption of light takes place as a result of transfer of energy and the output signal demonstrates a sharp dip at a particular wavelength known as resonance wavelength. The so-called resonance condition is given by following expression [6,7]:

(1) $$K_{0}n_{c}\text{sin}\vartheta =K_{0}{\left(\frac{\varepsilon_{\mathrm{mr}}n_{s}^{2}}{\varepsilon_{\mathrm{mr}}+n_{s}^{2}}\right)}^{1/2};\;K_{0}=\frac{2\pi }{\lambda }$$

The term on the left-hand side is the propagation constant (K_inc_) of the evanescent wave generated as a result of Attenuated Total Reflection (ATR) of the light incident at an angle θ through a lightcoupling device (such as prism or optical fiber) of refractive index n_c_. The right-hand term is the SPW propagation constant (K_SP_), with ε_mr_ as the real part of the metal dielectric constant (ε_m_) and n_s_ as the refractive index of the sensing (dielectric) layer. This matching condition of propagation constants is heavily sensitive to even a slight change in the outer ambience, which makes this technique a powerful tool for sensing of different parameters.

The performance of SPR sensors is analyzed with the help of three parameters: sensitivity, signal-to-noise ratio (SNR), and resolution.

In SPR sensors with spectral interrogation, the resonance wavelength (λ_res_) is determined with reference to the refractive index of the sensing layer (n_s_). If the refractive index of the sensing layer is altered by δn_s_, the resonance wavelength shifts by δλ_res_. The sensitivity (S_n_) of an SPR sensor with spectral interrogation is defined as:

(2) $$S_{n}=\frac{\delta \lambda_{\mathrm{res}}}{\delta n_{s}}\;\left(\text{nm}/\text{RIU}\right)$$

In other words, the sensitivity (S_n_) can be defined by calculating the shift in resonance wavelength per unit change in refractive index (nm/RIU). The Signal-to-Noise Ratio of an SPR sensor depends on how accurately and precisely the sensor can detect the resonance wavelength and hence, the refractive index of the sensing layer. This accuracy in detecting the resonance wavelength further depends on the width of the SPR curve.

The narrower the SPR curve, the higher the detection accuracy. Therefore, if δλ_SW_ is the spectral width of the SPR response curve corresponding to some reference level of transmitted power, the detection accuracy of the sensor can be assumed to be inversely proportional to δλ_SW_. The signal-to-noise ratio of the SPR sensor with spectral interrogation is, thus, defined as:

(3) $$\mathrm{SNR}\left(n\right)={\left[\frac{\delta \lambda_{\mathrm{res}}}{\delta \lambda_{\mathrm{SW}}}\right]}_{n}$$

where δλ_SW_ can be calculated as the full width at half maximum of the SPR curve (FWHM). SNR is a dimensionless parameter strongly dependent on the refractive index changes. The resolution (Δn) of the SPR-based optical sensor can be defined as the minimum amount of change in refractive index detectable by the sensor. This parameter definitely depends on the spectral resolution (δλ_DR_) of the spectrometer used to measure the resonance wavelength in a sensor scheme. Therefore, if there is a shift of δλ_res_ in resonance wavelength corresponding to a refractive index change of δn_s_, then resolution can be defined as:

(4) $$\Delta n=\frac{\delta n_{s}}{\delta \lambda_{\mathrm{res}}}\delta \lambda_{\mathrm{DR}}$$

### 2.2. Optical Sensor System and Experimental Setup

#### 2.2.1. Sample Fabrication

The fabricated optical sensor system was realized removing the cladding of a plastic optical fiber along half the circumference, spin coating on the exposed core a buffer of Microposit S1813 photoresist, and finally sputtering a thin gold film using a sputtering machine [8].

The plastic optical fiber has a PMMA core of 980 μm and a fluorinated polymer cladding of 20 μm. The refractive index, in the visible range of interest, is about 1.49 for PMMA, 1.41 for fluorinated polymer and 1.61 for Microposit S1813 photoresist. The sample consisted in a plastic optical fiber without jacket embedded in a resin block, with the purpose of easing the polishing process. The polishing process was carried out with a 5 μm polishing paper in order to remove the cladding and part of the core. After 20 complete strokes with a “Figure 8” pattern in order to completely expose the core, a 1 μm polishing paper was used for another 20 complete strokes with a “Figure 8” pattern. The realized sensing region was about 10 mm in length.

The buffer of Microposit S1813 photoresist was realized by using a spin coating. The Microposit S1813 photoresist is deposited in one drop (about 0.1 mL) on the center of the substrate. The sample is then spun at 6,000 rpm for 60 seconds. The final thickness of photoresist buffer was about 1.5 μm.

Finally, a thin gold film was sputtered by using a sputtering machine (Bal-Tec SCD 500).The sputtering process was repeated twice with a current of 60 mA for a time of 35 seconds (20 nm for step). The gold film so obtained was 40 nm thick and presented a good adhesion to the substrate, verified by its resistance to rinsing in de-ionized water.

#### 2.2.2. Experimental Setup

**Figure 1.**
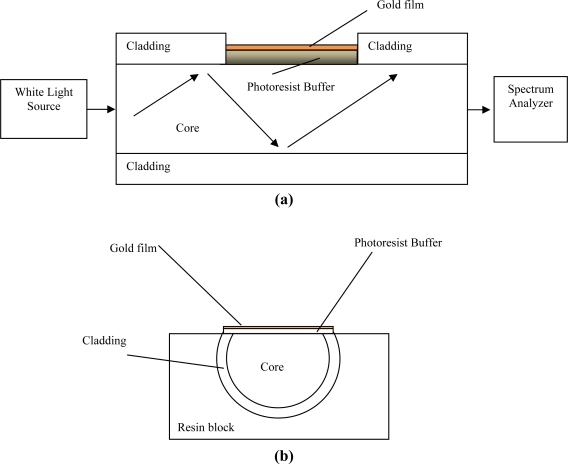
**(a)** Detail of sensor geometry and experimental; **(b)** Section of the system sensor.

The experimental setup was arranged to measure the transmitted light spectrum and was characterized by a halogen lamp, illuminating the optical sensor system, and a spectrum analyzer, as shown in Figure 1. The employed halogen lamp exhibits a wavelength emission range from 360 nm to 1,700 nm, while the spectrum analyzer detection range was from 200 nm to 850 nm. An Ocean Optics USB2000+UV-VIS spectrometer has been employed. The spectral resolution (δλ_DR_) of the spectrometer was 1.5 nm (FWHM). The spectrometer is finally connected to a computer. The SPR curves along with data values were displayed online on the computer screen and saved with the help of advanced software provided by Ocean Optics.

## 3. Results and Discussion

**Figure 2.**
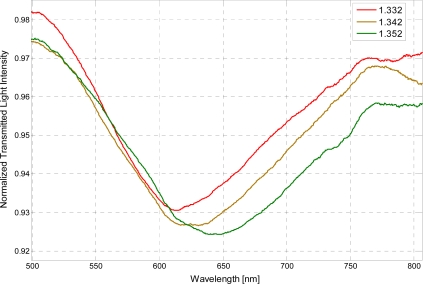
Experimentally obtained SPR transmission spectra, normalized to the air spectrum, for different refractive index of the aqueous medium. Configuration without the photoresist buffer layer.

In the series of performed experiments, water-glycerin solutions were used to achieve an aqueous medium with variable refractive index. For the sake of completeness, two configurations were tested, with and without the photoresist buffer layer. Without the buffer layer, in the same operating conditions, the sensor is capable of monitoring refractive indexes ranging from 1.33 to 1.36. In Figure 2 are presented the experimentally obtained SPR transmission spectra, normalized to the spectrum achieved with air as the surrounding medium [9,10], for three different water-glycerin solutions with refractive index ranging from 1.332 to 1.352.

**Figure 3.**
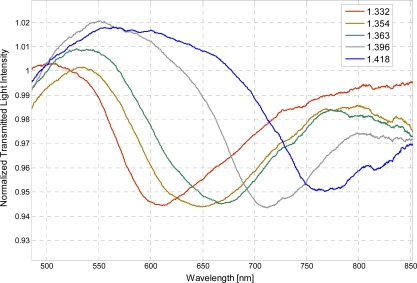
Experimentally obtained SPR transmission spectra, normalized to the air spectrum, for different refractive index of the aqueous medium. Configuration with the photoresist buffer layer.

In the presence of the photoresist buffer layer, the refractive index range is increased. In particular, this fiber optic sensor is capable of monitoring an aqueous environment whose refractive index range from 1.332 to 1.418. In Figure 3 are presented the experimentally obtained SPR transmission spectra, normalized to the spectrum achieved with air as the surrounding medium, obtained in this case with the photoresist buffer layer, for different water-glycerin solutions with refractive index ranging from 1.332 to 1.418.

**Figure 4.**
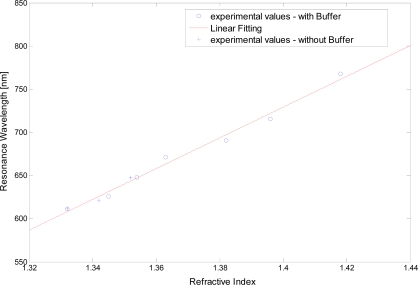
Plasmon resonance wavelength as a function of the refractive index. Configurations with and without the photoresist buffer layer.

Figure 4 shows the resonance wavelength *versus* the refractive index, for seven refractive index values (circles), obtained with the photoresist buffer layer, and for three refractive index values (crosses), obtained without the photoresist buffer layer.

In the same figure is also presented the linear fitting to the experimental data, showing a good linearity for the sensors with and without the photoresist buffer layer. The Pearson’s correlation coefficient is 0.9543 for the sensor without buffer layer and 0.9918 for the sensor with buffer layer.

**Figure 5.**
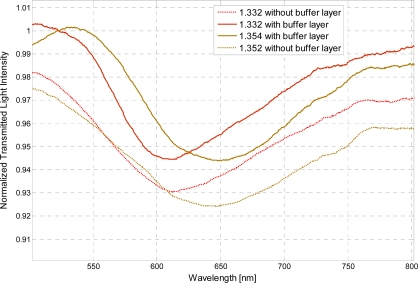
The shift in resonance wavelength with a change in refractive index of the aqueous medium for the two configurations tested, with and without the photoresist buffer layer.

**Figure 6.**
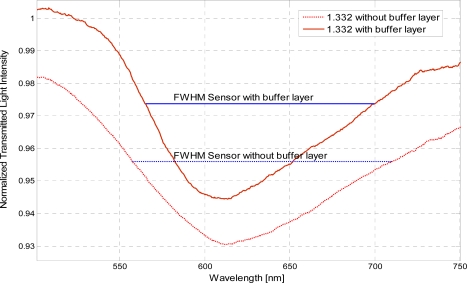
The full width at half maximum (FWHM) of the SPR curve for the two sensors configurations with and without the buffer layer for an external refractive index of 1.332.

In presence of the photoresist buffer layer, the refractive index range is increased while the sensitivity is the same. Figure 5 shows the shift in the resonance wavelength and the width of the resonance curve corresponding to a change in refractive index of the aqueous medium for the two configurations tested. Without the photoresist buffer layer there is a decrease in the power transmitted to the fiber end facet, due to a greater dissipation. This decrease results in the decrease of the SPR curve and the increase of the SPR curve width, as shown in Figure 5. Therefore, it can be conveniently established that SPR curve width increases (δλ_SW_) without the photoresist buffer layer, as shown for example in Figure 6 for a refractive index equal to 1.332.

The observed absorption band is the result of the convolution of different resonance peaks. Each peak is obtained for a specific resonance condition defined by a given angle-wavelength couple [6].

For a comparative analysis between sensors with buffer and without buffer layer, Tables 1 and 2 report the experimentally measured performance parameters, evaluated by Matlab software, at a refractive index of 1.354 with the buffer layer and 1.352 without the buffer layer, respectively. It can be stated that with the photoresist buffer layer the SNR and the sensitivity are increased. Note that the reported SNRs are larger than those reported in Reference [6], showing a better performance for the proposed sensor.

The ultimate sensitivity of the realized optical sensor depends on the sensing region length, on the thickness of the photoresist buffer and on the thickness of the gold film, as well [6,11]. For the comparative analysis the realized sensors have been tested for two weeks showing good reproducibility and reversibility of the process.

## 4. Conclusions

Two plastic optical fiber based SPR sensor configurations, useful for biosensing applications, have been realized and experimentally tested. The proposed devices are based on the excitation of surface plasmons at the interface between under test medium and a thin gold layer deposited on a photoresist buffer spin coated on the plastic fiber core or directly on the fiber core. The sensing devices have been characterized by exploiting a halogen lamp to illuminate the optical fiber and observing the transmitted spectra, normalized to the spectrum transmitted when the outer medium is air. The experimental results indicate that the configuration with the photoresist buffer layer exhibits better performance in terms of detectable refractive index range and SNR.

## Figures and Tables

**Table 1.** Performance parameters for the sensor with the buffer layer at an external refractive index of 1.354.

**Refractive index**	**Resolution (Δn) [RIU]**	**Resonance wavelength (λ_res_) [nm]**	**Signal-to-noise ratio (SNR)**	**Sensitivity (S_n_) [nm/RIU]**	**Full width at half maximum of the SPR curve (FWHM) [nm]**
1.354	0.00059	647.8	2.329	2.533 × 10^3^	151

**Table 2.** Performance parameters for the sensor without the buffer layer at an external refractive index of 1.352.

**Refractive index**	**Resolution (Δn) [RIU]**	**Resonance wavelength (λ_res_) [nm]**	**Signal-to-noise ratio (SNR)**	**Sensitivity (S_n_) [nm/RIU]**	**Full width at half maximum of the SPR curve (FWHM) [nm]**
1.352	0.00062	643.81	0.982	2.422 × 10^3^	181

## References

[1] Anuj K., Sharma R.J., Gupta B.D. (2007). Fiber-Optic Sensors Based on Surface Plasmon Resonance: A Comprehensive Review. IEEE Sensors J.

[2] Bartlett R.J., Philip-Chandy R., Eldridge P., Merchand D.F., Morgan R., Scully P.J. (2000). Plastic Optical Fibre Sensors and Devices. Trans. Inst. Meas. Control.

[3] Piliarik M., Homola J., Manikova Z., Čtyroký J. (2003). Surface Plasmon Resonance Sensor Based on a Single-Mode Polarization-Maintaining Optical Fiber. Sens. Actuat. B Chem.

[4] Lomer M., Arrue J., Jauregui C., Aiestaran P., Zubia J., Lopez-Higuera J.M. (2007). Lateral Polishing of Bends in Plastic Optical Fibres Applied to a Multipoint Liquid-Level Measurement Sensor. Sens. Actuat. A Phys.

[5] Muñoz-Berti V.M., López-Pérez A.C., Alén B., Costa-Krämerm J.L., García-Martín A., Lomer M., López-Higuera J.M. (2010). Low Cost Plastic Optical Fiber Sensor Based on Surface Plasmon Resonance. Proc. SPIE.

[6] Kanso M., Cuenot S., Louarn G. (2008). Sensitivity of Optical Fiber Sensor Based on Surface Plasmon Resonance: Modeling and Experiments. Plasmonics.

[7] Dwivedi Y.S., Sharma A.K., Gupta B.D. (2008). Influence of Design Parameters on the Performance of a SPR Based Fiber Optic Sensor. Plasmonics.

[8] Cennamo N., Massarotti D., Conte L., Zeni L. SPR in Plastic Optical Fibers: A Simple Geometry for Low-Cost Biosensors.

[9] Sipe J.E. (1979). The ATR Spectra of Multipole Surface Plasmons. Surf. Sci.

[10] Roy D. (2001). Surface Plasmon Resonance Spectroscopy of Dielectric Coated Gold and Silver Films on Supporting Metal Layers: Reflectivity Formulas in the Kretschmann Formalism. Appl. Spectrosc.

[11] Iga M., Seki A., Watanabe K. (2005). Gold Thickness Dependence of SPR-Based Hetero-Core Structured Optical Fiber Sensor. Sens. Actuat. B Chem.

